# New Challenges of HIV-1 Infection: How HIV-1 Attacks and Resides in the Central Nervous System

**DOI:** 10.3390/cells8101245

**Published:** 2019-10-13

**Authors:** Victoria Rojas-Celis, Fernando Valiente-Echeverría, Ricardo Soto-Rifo, Daniela Toro-Ascuy

**Affiliations:** 1 Instituto de Ciencias Biomedicas, Facultad de Ciencias de la Salud, Universidad Autónoma de Chile, Santiago 8910060, Chile; victoria.rojcel@gmail.com; 2 Molecular and Cellular Virology Laboratory, Virology Program, Institute of Biomedical Sciences, Faculty of Medicine, Universidad of Chile, Santiago 8389100, Chile; fvaliente@med.uchile.cl (F.V.-E.); rsotorifo@med.uchile.cl (R.S.-R.)

**Keywords:** AIDS, HIV-1, HAND, CNS, cART, CNS cells

## Abstract

Acquired immunodeficiency syndrome (AIDS) has become one of the most devastating pandemics in recorded history. The main causal agent of AIDS is the human immunodeficiency virus (HIV), which infects various cell types of the immune system that express the CD4 receptor on their surfaces. Today, combined antiretroviral therapy (cART) is the standard treatment for all people with HIV; although it has improved the quality of life of people living with HIV (PLWH), it cannot eliminate the latent reservoir of the virus. Therefore HIV/AIDS has turned from a fatal disease to a chronic disease requiring lifelong treatment. Despite significant viral load suppression, it has been observed that at least half of patients under cART present HIV-associated neurocognitive disorders (HAND), which have been related to HIV-1 infection and replication in the central nervous system (CNS). Several studies have focused on elucidating the mechanism by which HIV-1 can invade the CNS and how it can generate the effects seen in HAND. This review summarizes the research on HIV-1 and its interaction with the CNS with an emphasis on the generation of HAND, how the virus enters the CNS, the relationship between HIV-1 and cells of the CNS, and the effect of cART on these cells.

## 1. Introduction

Human immunodeficiency virus (HIV)/acquired immunodeficiency syndrome (AIDS) is a major public health problem worldwide. Nearly 38 million people are currently infected with the virus, and an average of one million people die every year from AIDS-related illnesses [1]. In the mid-1990s, the development of inhibitors of the viral reverse transcriptase and protease, two of the crucial enzymes required for the replication of HIV-1 and their administration in combination—known as combined antiretroviral therapy (cART)—represented a breakthrough in the fight against AIDS, considerably increasing the survival of people living with HIV (PLWH) [2]. With the decrease in mortality, HIV/AIDS was transformed from a fatal disease to a chronic disease. However, this has led to problems related with chronic inflammation; at least 50% of PLWH have chronic problems in this respect, even with cART administration [3]. Although cART can reduce the viral load and number of opportunistic infections, it does not eliminate the virus present in the latent reservoirs. Therefore, with the increase in life expectancy, HIV-positive patients have seen an increase in neurocognitive dysfunction associated with HIV-1 [4]. Although not all the neurocognitive dysfunction disorders manifest with apparent symptoms, the accumulation of these symptoms can significantly decrease the quality of life of PLWH. Thus, the search for treatments that can eliminate the latent HIV-1 reservoirs and/or counteract the adverse effects on the central nervous system (CNS) is crucial. This review summarizes the research on HIV-1 and its interaction with the CNS with emphasis on i) the generation of HIV-associated neurocognitive disorders; ii) how the virus enters the CNS; iii) the relationship between HIV-1 and cells of the CNS; and iv) the effect of cART on these cells.

## 2. HIV-1 Replication Cycle

HIV-1 is an important infectious agent that is responsible for AIDS [5]. The HIV-1 infectious particle consists of a host cell-derived lipid bilayer, which contains the viral envelope glycoprotein gp120 and the transmembrane protein gp41. Under the envelope and attached to lies a spherical protein shell known as matrix. Inside the particle is the capsid that contains two identical copies of the viral RNA and viral proteins necessary for the replication of the virus including the enzymes reverse transcriptase, integrase and protease [6]. HIV-1 has tropism toward cells that express the CD4 receptor on their surfaces. Therefore, it can infect T lymphocytes [7], monocytes/macrophages [8], dendritic cells [9], and microglia [10]. The life cycle of HIV-1 begins with the interaction between the glycoprotein gp120 on the virus surface and the N-terminal extracellular domain of the CD4 receptor as well as one of the co-receptors, CCR5 or CXCR4 [11]. The formation of this complex leads to conformational changes in glycoprotein gp41, which triggers membrane fusion and the subsequent entry of the viral capsid into the host cell cytoplasm [12]. Upon uncoating, the capsid disappears but at least some matrix, nucleocapsid, reverse transcriptase, and integrase proteins, and the accessory protein Vpr, remain associated to convert the viral genome to a double-stranded DNA, which is transported to the nucleus as part of a pre-integration complex [13]. In the nucleus, the integrase catalyzes the insertion of the linear double-stranded viral DNA into the host cell chromosome, creating a provirus [14]. This provirus serves as a template for the synthesis of viral RNAs that encode structural proteins (Gag and Env), enzymes (reverse transcriptase, polymerase, and integrase) as well as regulatory and accessory proteins (Tat, Rev, Nef, Vif, Vpu, and Vpr) [15]. Once integrated, the provirus can follow three paths depending of the cell type infected: i) remain latent; ii) replicate in a controlled manner; or iii) undergo massive replication, with a consequent cytopathic effect on the infected cell [16]. When the HIV-1 Tat protein is present, transcription can increase by about 100- to 1000-fold. The viral mRNA is synthesized in the form of a 9-kb single transcript similar to the viral genome that entered the cell. In the early stages, the 9-kb RNA is processed into transcripts of different sizes and, in later stages, the complete viral RNA remains unprocessed and is transported to the cytoplasm to be used as the mRNA for the synthesis of the structural protein Gag [17]. The viral protein Rev is necessary for the transport of the complete viral mRNA outside the nucleus, and for the association of its mRNA targets with the host translational machinery [18]. Once the levels of Gag reach a certain threshold, and the synthesis of all complementary viral proteins occurs, the virus is assembled at the plasma membrane, where Env interacts with Gag, and newly produced viruses are released to begin a new replication cycle. The final maturation of virions and the correct assembly of the viral proteins occur at the last step of the infective cycle, during the release of the viruses through the cell membrane, allowing mature viral particles to be produced [19].

## 3. HIV-Associated Neurocognitive Disorder (HAND)

A few decades ago, with the introduction of cART and the increase in the life expectancy of HIV patients, signs and symptoms indicating a decline of brain function and movement capacity as well as changes in behavior and mood were observed, exposing a state known as HIV-associated neurocognitive disorder (HAND) [20]. According to the severity of the clinical manifestations, HAND is separated into three conditions: asymptomatic neurocognitive impairment (ANI), mild neurocognitive disorder (MND), and HIV-associated dementia (HAD) [20]. Neurocognitive testing on PLWH should include assessment of at least five features, such as attention, information processing, language, abstraction/executive functioning, sensory-perceptual skills, simple motor skills, complex perceptual-motor skills, or memory (including learning and recall). The definition for ANI was recently adopted by Frascati in 2007 [21]. Generally, diagnosing ANI is complex, as the deterioration of cognitive functions is mild. The cognitive-behavioral disorders associated with ANI can involve mild motor slowdown (which is registered by the patient but barely noticeable to others) or slight difficulty remembering. However, associating these small changes with HIV-1 infection is difficult [21]. Despite this, research indicates that ANI is clinically important to detect since it can rapidly evolve to a severe form of HAND [22]. The definition of MND is very similar to that of ANI. However, in MND, the decline of cognitive function slightly interferes with the daily life of the HIV-infected patient, affecting their mental acuity and decreasing their effectiveness at work, in their domestic life, and social life. These changes are noticed both by the patients experiencing changes and by the people around them [20]. Finally, HAD, the most severe of the HAND disorders, is the best known as its effects were identified before the cART era. HAD is characterized by an increase in the loss of attention and concentration, notable motor slowing, and various behavioral components, and it generally leads to death within one year [23]. This syndrome is associated with pathological changes in the brain that include generalized atrophy, changes in white matter causing leukoencephalopathy, microglial nodules typical of viral encephalitis, and multinucleated giant cells that seem to be infected directly by HIV-1 (based on antigen staining) [24]. The arrival of cART reduced the incidence of HAD in PLWH by 40–50% [25].

To diagnose any of these three HAND states, it is crucial to rule any evidence of any other cause that may be inducing the neurocognitive deterioration. It is also necessary to consider the risk factors that can influence the severity of the HAND diagnosis. The most important risk factors are age, disease phase, and viremia [26]. Risk factors may also include low educational level [27], low level of CD4 lymphocytes, sexual orientation [28,29], and coinfection with other viruses such as hepatitis C virus [30]. Additionally, several chronic diseases can contribute to neurocognitive dysfunction such as cardiovascular diseases [31], diabetes, and other metabolic problems [32]. The high depression rate (70%) among HIV-positive patients inherently contributes to neurological damage in the population [33]. The use of drugs such as methamphetamines [34], cannabis [35], and cocaine [36] is another risk factor for HAND. All these factors must be considered when diagnosing any category of HAND. In many cases, the use of additional criteria is necessary to obtain a more accurate diagnosis.

## 4. How Does HIV-1 Enter the CNS?

HIV-1 infection is known to induce a decrease of the population of CD4^+^ T lymphocytes. However, HIV-1 can also act on the CNS tissues, for example, the brain, spinal cord, and peripheral nerves [37]. Pathological anatomy research has demonstrated the presence of viral particles in astrocytes, microglia, oligodendrocytes, and in a lower proportion, neurons [38]. The effect of HIV-1 on the CNS depends on the HIV-1 infection stage. For example, in the early stage (CD4^+^ count: >500 cells/µL), neurologic complications are due to the virus infection or to the action of multiple biological processes mediated by the immune system. In the intermediate stage (CD4^+^ count: 201–500 cells/µL), the adverse effects are the result of the indirect actions of the immune system and the metabolic effects of antiretroviral drugs. Finally, in the last stage (CD4^+^ count: ≤200 cells/µL), neurological complications are the result of the previously mentioned factors in conjunction with the action of opportunistic infections and/or tumors [39].

It is well known that the entry of HIV-1 into the CNS occurs in the first week (or weeks) after infection [40]. Although the entry mechanism has not yet been clarified, certain theories have been suggested. One of them is the “Trojan horse” theory, which proposes that the virus enters the CNS through monocytes or infected CD4^+^ T lymphocytes [41]. Other studies have shown that HIV-1 can alter the permeability of the blood–brain barrier (BBB) by modifying the expression of proteins that participate in maintaining BBB epithelial tight junctions through the action of the viral protein Tat [42,43,44]. Another theory proposes that viral particles freely travel to the CNS. In the case of HIV-1, particles can cross the BBB by transcytosis, which is mediated by the viral envelope protein gp120 [45]. This process can also occur when the BBB is altered by an increase in proinflammatory cytokines, as demonstrated by a study in which the presence of the HIV-1 p24 antigen in the brain was detected only after the disruption of the BBB by tumor necrosis factor (TNF)-α and interleukin (IL)-6 [46]. Another proposed route of entry is through infected epithelial cells. This theory is related to the “Trojan horse” theory, as epithelial cells in HIV-infected individuals increase the expression of adhesion molecules such as E-selectin either due to HIV-1 infection [47] or due to the presence of HIV-infected circulating monocytes [48].

When HIV-1 is established in lymphoid nodules, it can amplify its production as a result of the interaction between infected T lymphocytes and circulating dendritic cells [49]. This environment is ideal for virus replication because the increase in proinflammatory cytokines (such as IL-1, IL-6, and TNF-α) promotes viral replication [50]. Once inside the CNS, HIV-1 can cause progressive neurotoxicity, neurodegeneration, and a chronic activation of the inflammatory response. This damage may also be a consequence of immunosuppression, which in turn, is responsible for opportunistic infections and neoplasms that affect the brain [51].

## 5. Cells Infected by HIV-1 in the CNS

### 5.1. HIV-1 Infection in Microglia

Microglial cells belong to the innate immune system and are the resident tissue macrophages of the CNS [52]. Microglia have multiple functions, including support of CNS development and synaptogenesis and participation in the immune response against infectious agents, neuroinflammation, and degenerative diseases [52]. Microglia are, therefore, the key cell population linking both the nervous and immune systems. Consequently, the hyperactivation of microglia has been associated with the development of various neurological disorders, including Alzheimer’s [53], Huntington’s [54], and Parkinson’s diseases [55], as well as HAND [56].

The entry of HIV-1 into a cell requires not only the presence of CD4 but also the CXCR4 or CCR5 coreceptors. However, in the CNS, CCR3 and CCR5 are also required to promote efficient infection. Microglia are susceptible to infection as they have both co-receptors (CCR3 and CCR5) on their surfaces [57], with CCR5 more strongly associated with virus entry and subsequent development of dementia [58]. Microglia together with perivascular macrophages, are the perfect candidates for the virus to remain latent as they are renewed over months or years [59]. Several latency mechanisms in microglia have been proposed. Kumar et al. used a microglial cell line to establish that the high mobility group AT-hook 1 (HMGA1) protein is recruited to an inactive complex formed by COUP Transcription Factor (COUP-TF) interacting protein 2 (CTIP-2) and the Positive Transcription Elongation Factor (P-TEFb) on the HIV-1 long terminal repeat (LTR) [60]. In another study, using human fetal microglia stimulated with interferon (IFN)-α and infected with HIV, the virus was not able to produce viral RNA and DNA and the viral spread was restricted due to the induction of tetherin expression, a host restriction factor [61].

Microglial activation helps to protect the CNS from viruses and pathogens. However, their overactivation and/or chronic activation can lead to neuronal damage. The contribution of microglia to cognitive deterioration in the different forms of HAND involves the increase in proinflammatory chemokines and cytokines, in addition to the increase in neurotoxins that affect both astrocytes and neurons and can induce neuronal apoptosis [62]. For example, in neurological tissue from HIV-infected patients with signs associated with dementia, an increase in TNF-α mRNA levels in microglia and astrocytes has been reported [63].

Similar to what is observed during aging, HIV-1 infection can generate an inflammatory environment by causing the release of viral proteins (such as Tat and gp120) and cellular products (such as proinflammatory cytokines, e.g., TNF-α, IL-8, IL-6, and IL-1β) from cells [64]. Recent studies have shown that in microglia, the HIV-1 Tat protein can cause activation of the Nod-like receptor (NLR) family pyrin domain containing 3 (NLRP3) inflammasomes. This translates into an increase in caspase-1 and IL-1β levels, which in turn induces the production of TNF-α and IL-6, exacerbating the proinflammatory environment [65]. Likewise, HIV-1 infection in microglia can cause the production of reactive oxygen species (ROS) and reactive nitrogen species (RNS) such as quinolinic acid, arachidonic acid, and nitric oxide [66]. Moreover, HIV-1 can induce the expression of inducible hypoxia factor (HIF)-1, causing mitochondrial dysfunction and oxidative stress [67]. Another study showed that HIV-1 infection in primary human microglia induced the secretion of IL-6, IL-8, TNF-α, C-C Motif Chemokine Ligand 2 (CCL2), and Regulated upon Activation, Normal T cell Expressed and Secreted protein (RANTES) [68]. The same study showed that HIV-1 infection induces autophagy activation that is dependent on beclin 1, which is correlated with the release of the viral protein p24 and the production of proinflammatory cytokines. Hence, HIV-1 replication in microglia and the activation of these cells are dependent on autophagy activation [68].

Despite the critical relevance of microglia infection in HAND, studies that characterize the replication of HIV-1 in these cells are scarce as they involve using primary cultures obtained from fetal brains or post-mortem assessment of AIDS patients, which are both difficult to access [69]. Therefore, the mechanism of HIV-1 action in microglia remains largely unknown. In 2017, Garcia-Mesa et al. developed an immortalized human microglial cell line, which has microglia-like morphology, expresses key microglial surface markers, has appropriate migratory and phagocytic activity, and has the capacity to establish an inflammatory response characteristic of primary microglia. They showed that these cells were useful to generate stable cell lines latently infected with HIV-1 proviruses. This well-characterized cell line represents an invaluable tool for studying microglial cell function and the mechanics and dynamics of HIV-1 replication in the CNS [70].

### 5.2. HIV-1 Infection in Perivascular Macrophages

Perivascular macrophages correspond to resident cells of the immune system in the brain, specifically in the perivascular space. They are a structural part of the BBB, playing an essential role in the communication between the immune system and the brain, transmitting and modulating peripheral inflammatory signals [71]. Similar to microglia, macrophages are susceptible to be infected by HIV-1 as they are CD4^+^ and express the CXCR4 and CCR5 coreceptors. However, the most used coreceptor is CCR5 as evidence suggests that HIV-1 particles entering macrophages by CXCR4 have no infective capacity [72].

HIV-1 can also cause genomic instability in macrophages, as macrophages can be induced to produce ROS and nitric oxide, which interfere with the signaling pathways related to apoptosis and cell cycle arrest and can cause severe damage to DNA and proteins [73].

A study carried out in 2001 by Lane et al. showed that the cerebrospinal fluid (CSF) of patients with HAD had high levels of proinflammatory cytokines, including IL-8, which increases HIV-1 replication in T cells and macrophages [74,75]. Additionally, macrophages infected with HIV-1 produce neuronal signaling products; for example, they release adenosine triphosphate (ATP), resulting in neurotoxic levels of glutamate [76].

### 5.3. HIV-1 Infection in Astrocytes

Astrocytes are the primary and most abundant glial cells of the CNS. They participate in homeostasis and structural support of neurons in addition to contributing to proper nervous system functioning by inactivating neurotransmitters and maintaining extracellular potassium levels [77].

Interestingly, although astrocytes do not express CD4 or CCR5, it is possible detect HIV-1 as infectious virions, viral proteins, and even nucleic acids in cultured astrocytes [78]. A study made by Russell et al. showed that HIV-1 particles can enter by endocytosis in a primary cell culture from human fetal astrocyte, but without integrate into the host’s DNA. Moreover, the same authors demonstrated that astrocytes can engulf fragments of HIV-1-infected macrophages via ‘eat me’ [79]. This process would explain the presence of viral DNA even in the absence of infection. On the other hand, Carroll-Anzinger et al. showed that in the presence of pro-inflammatory stimuli like interferon-γ, astrocytes can be infected [80]. Nevertheless, unlike in microglia and macrophages, HIV-1 replication in astrocytes is restricted [81] and HIV-1 can remain latent due to silencing at the epigenetic level of the viral DNA [82].

An essential feature of astrocytes is that they are unable to produce complete viral particles. However, they may contribute to HIV-related brain damage due to the generation of astrogliosis [83]. In this process, the glial scar is formed, which inhibits axonal regeneration and cell migration, which directly affects neuronal development. In this same context, Bansal et al. induced astrogliosis through injection of viral protein gp120 into the corpus striatum of rats [84]. Astrocytes can also produce and release several viral proteins such as Tat, Nef, and Rev, which promote inflammation and, therefore, neuronal damage [85]. For example, Tat is known to activate HIV-1 transcription, and it can also enhance HIV-1 infection of primary human astrocytes [86], indicating that astrocytes could play a fundamental role in the spread of HIV-1 in the CNS. On the other hand, Nef can induce ROS production in human astrocytes which promotes rapid neuronal death, thus contributing to the development of HAD [67]. This effect of Nef could explain why patients who do not adhere to or do not receive cART have a higher risk of developing dementia in the short term.

Astrocytes infected with HIV-1 can also alter the BBB. A study by Eugenin et al. showed that the infection of a small percentage of astrocytes is enough to produce alterations in the BBB due to epithelial cell apoptosis (which is explained by the ability of astrocytes to release Tat), misguided astrocyte endfeet and dysregulation of lipoxygenase/cyclooxygenase, large-conductance Ca2+- and voltage-gated big K+ (BK_Ca_) channels, and ATP receptor activation in astrocytes [87]. Astrocytes infected can also produce changes in microglia, because infection induces astrocytes to release microRNA-9 (miR-9), which is uptaken by the microglia. Inside microglia, miR-9 can regulate the expression of genes related to migratory phenotype; this process may contribute to HIV-induced neuropathogenesis [88].

### 5.4. HIV-1 Infection in Oligodendrocytes

Oligodendrocytes are glial cells that myelinate neuronal axons. The difficulty in isolating oligodendrocytes makes it challenging to establish a cell line to determine whether HIV-1 can infect them. Oligodendrocytes are CD4 and CCR5 negative but express CXCR4 [89]. It is thought that most of the damage observed in oligodendrocytes is due to the release of viral proteins from other infected cells [90,91]. Zou et al. identified that the Tat protein causes oligodendrocyte death or immaturity and reduces myelin-like membranes in mature oligodendrocytes. This effect could be explained by the interaction of Tat with the N-methyl-D-aspartate (NMDA) receptor, which induces an increase in Ca^2+^ and Ca^2+^/calmodulin-dependent protein kinase II (CaMKIIβ) [92]. Additionally, a recent study showed that damage in oligodendrocytes produced by Tat is triggered by the altered balance of CaMKIIβ and glycogen synthase kinase 3β (GSK3β); this interaction is implicated in oligodendrocyte apoptosis as well as HIV-1 neuropathology [93].

### 5.5. HIV-1 Infection in Neurons

Neurons are the basic functional units of the nervous system. They are electrically excitable cells that process and transmit information. The infective capacity of HIV-1 in neurons is still a matter of debate. However, some researchers have managed to infect immature neurons [94]. Adult neurons express CXCR4, CCR5, and CCR3 on their surfaces [95,96]. CD4 has also been visualized in cerebellar, thalamic, pons, and hippocampal neurons [97], but other studies indicate the absence of this receptor [98]. However, the presence of CCR3 suggests that HIV-1 could enter into neurons using a CD4-independent mechanism.

Regardless of whether HIV-1 enters the neurons, viral proteins from other CNS cells can cause a detrimental effect on neuronal functioning, even causing neuronal death. For example, it is known that the Tat protein can interact with phagosome markers in neurons, altering the phagosome morphology and preventing phagosome binding to lysosomes. Thus, neurons are prevented from degrading foreign proteins such as HIV-1 proteins [99]. A similar effect occurs with the viral protein Nef, which prevents autophagic maturation contributing to neurodegeneration [100]. In addition, Tat can induce neuronal apoptosis by activating forkhead box O3 (FOXO3), which is translocated to the nucleus through c-Jun N-terminal kinase (JNK) signaling to upregulate the expression of pro-apoptotic genes such as Bcl-2-like 11 (Bim) and downregulate anti-apoptotic factors such as B-cell lymphoma 2 (Bcl-2) [101].

## 6. Effect of cART in the CNS and Possible Treatments for HIV-1

Today, cART is the standard treatment for all people newly diagnosed with HIV-1. cART has largely improved the quality of life of infected people, reducing morbidity and mortality associated to HIV/AIDS. Indeed, cART transformed HIV/AIDS from a mortal disease to a chronic disease requiring lifelong treatment. However, cART still faces important challenges such as its inability to eliminate the latent reservoir or to control persistent replication in macrophages and microglia. Several studies have reported that once patients stop treatment, a resurgence of HIV-1 takes place, which derives from latent reservoirs or from cells with persistent replication [78,102,103]. In the last decade, it was determined that the increase in the new cases of neurocognitive disorders in HIV-1 positive patients is due to the HIV-1 infection itself and not an infection by other opportunistic pathogens [104,105,106].

Currently, there is not a cure for HAND and the only treatment involves strict compliance with antiretroviral therapy to keep a low viral load in the blood [107]. Although cART is unable to eliminate the latent provirus, it has been shown to help improve the brain function of PLWH [108]. However, today there is a great debate about the real effect of antiretroviral drugs on the CNS, as protective effects, as well as adverse effects of the use of therapy. Some studies show a reduction in cognitive impairment due to the use of cART. In contrast, patients with a controlled viral load who discontinue treatment with antiretrovirals show an improvement in cognitive functions and a reduction in neuronal damage [109,110,111]. The problems of therapy are largely related to the secondary effects of some antiretrovirals, such as efavirenz, stavudine, zidovudine and abacavir, which are associated with neurological disorders [112]. For example, efavirenz is associated with changes in sleep quality, anxiety, and depression during the first week of use [113]. Although these symptoms were only seen the first week of a 24-week treatment, this directly affects the adherence of patients to treatment. The development of a cure or strategy to fight the latent reservoir of HIV-1 has been very difficult. First by features of the virus, such as the high mutation rate and its ability to adapt to allow HIV-1 to enter or act on different target cell types [113]. Another important consideration is that the drugs reach different concentrations in the different cells of the CNS [107]. Furthermore, the concentration of the various drugs mixed in cART is dependent on the composition of the specific therapy used [113], which means that each drug may achieve different concentrations in the same cell, so optimal functionality of the drugs is difficult to ensure.

Recent research has focused on influencing inflammatory cascade, oxidative stress, neurotoxin release, viral replication, apoptosis, and neuroprotection [114]. For example, one study showed that adding two anti-inflammatory and immunosuppressive drugs (teriflunomide and monomethyl fumarate) to a co-culture of human monocytoid cells and human-activated microglia led to decreased levels of CCL2, CCL5, and CXCL10, which are proinflammatory cytokines related to neurotoxicity [115]. In addition to antiretrovirals, many other medications have been studied as adjuvants for the treatment of neurocognitive impairment associated with HIV-1 infection. For example, Mamik et al. developed an insulin treatment that in HIV-infected primary human microglia suppressed the HIV-1 p24 levels in the supernatant and reduced CXCL10 and IL-6 transcript levels [116]. The mechanisms underlying the effect of insulin may be related to a variety of metabolic and trophic effects and can directly protect neurons and dampen inflammatory cytokine expression [116]. This new treatment represents a new therapeutic option for patients with HAND.

## 7. Conclusions and Future Perspectives

**Figure 1:**
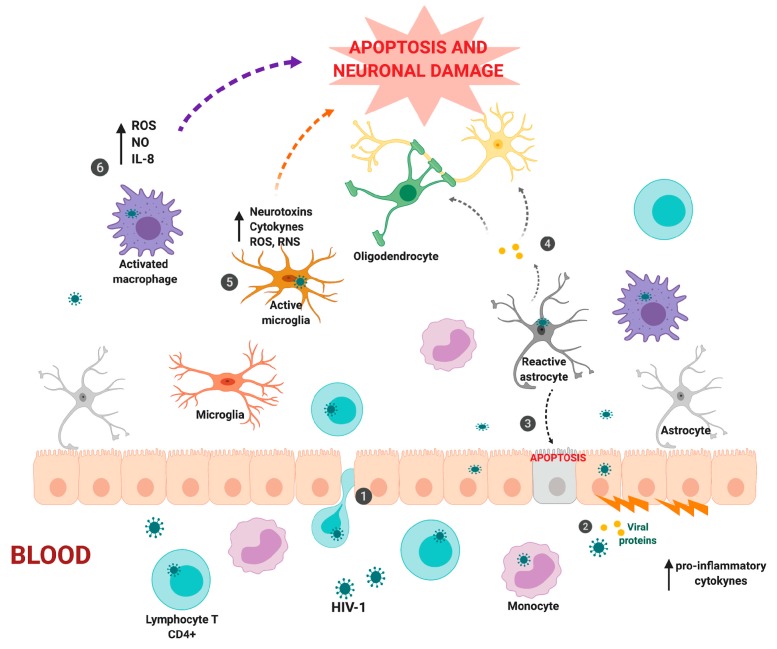
Entry of human immunodeficiency virus (HIV)-1 into the central nervous system and its effects on cells that lead to damage and death of neurons. (**1**) HIV-1 can enter through monocytes or infected T cells that migrate from the bloodstream to the central nervous system (CNS) (Trojan horse theory). (**2**) The increase in pro-inflammatory cytokines and viral proteins can alter the permeability of the epithelial cells of the blood–brain barrier, making virus entry easier. In addition, the virus can use infected epithelial cells and reach the other side through transcytosis. (**3**) Reactive astrocytes can induce epithelial cell apoptosis, contributing to the modification of the permeability of the blood–brain barrier through the release of viral proteins such as Tat. (**4**) The viral protein Tat has a direct effect on oligodendrocytes and neurons, which produce increased damage and neuronal death. Chronic activation of activated (**5**) microglia and (**6**) macrophages generates an increase in the levels of proinflammatory cytokines, neurotoxins, reactive oxygen species (ROS), and reactive nitrogen species (RNS) (created with BioRender).

It has been suggested that HIV-1 can enter the CNS in different ways; one way is the virus could enter through monocytes or infected T cells that migrate from the bloodstream to the CNS (Trojan horse theory). The increase in pro-inflammatory cytokines and viral proteins can alter the permeability of epithelial cells of the BBB, facilitating virus entry. Alternatively, it was proposed that the virus could use infected epithelial cells and reach the other side through transcytosis, or reactive astrocytes which induce epithelial cell apoptosis, contributing to the modification of the permeability of the blood–brain barrier through the release of viral proteins. On the other hand, the viral protein Tat, which has a direct effect on oligodendrocytes and neurons providing increased injury and neuronal death, can induce the damage caused by HIV-1 in the CNS. Also, it was reported that the chronic activation of activated microglia and macrophages generates an increase in the levels of proinflammatory cytokines, neurotoxins, ROS, and RNS (Figure 1).

In recent years, several studies have aimed to understand how HIV-1 damages CNS cells. The studies cited in this review analyze the effects of HIV-1 at the molecular level and how HIV-1 damages CNS cells. However, a link that defines a clear mechanism of how the virus triggers neurodegenerative diseases is not yet established. Research leads us to suggest that neuronal damage is likely caused by the pro-inflammatory and reactive environment that is generated as a result of all the inflammatory cytokines, ROS, and RNS that surround neurons. Notwithstanding, it is still necessary to study the mechanisms that regulate the viral latency and the signals involved in its reactivation.

Unfortunately, studies of infection by HIV-1 in CNS cells are difficult because many primary cell cultures from the CNS are not 100% pure, making it difficult to differentiate the cell types affected by the virus. Abud et al. established a protocol to differentiate pluripotent stem cells (iPSCs) from human microglial-like cells (iMGL), using different factors that mimic the environment present in embryonic development [117]. The authors showed by both transcriptomic and functional analyses that iMGLs are similar to cultured human adult and fetal microglia [117]. Recently, Tcw et al. performed a protocol to differentiate human induced pluripotent stem cells (hiPSCs) to astrocytes which were similar to primary human fetal astrocytes [118]. Both studies provide the basis to generate a model that will allow a better characterization of the infection by HIV-1.

Further research is necessary to elucidate the detail of how HIV-1 infection and spread operates in the CNS and how it may trigger neurodegenerative diseases. Proper determination of the mechanism of HIV-1 infection in the CNS is the first step in the development of new strategies to combat these non-AIDS diseases caused by HIV-1.
